# Common Variants Show Predicted Polygenic Effects on Height in the Tails of the Distribution, Except in Extremely Short Individuals

**DOI:** 10.1371/journal.pgen.1002439

**Published:** 2011-12-29

**Authors:** Yingleong Chan, Oddgeir L. Holmen, Andrew Dauber, Lars Vatten, Aki S. Havulinna, Frank Skorpen, Kirsti Kvaløy, Kaisa Silander, Thutrang T. Nguyen, Cristen Willer, Michael Boehnke, Markus Perola, Aarno Palotie, Veikko Salomaa, Kristian Hveem, Timothy M. Frayling, Joel N. Hirschhorn, Michael N. Weedon

**Affiliations:** 1 Department of Genetics, Harvard Medical School, Boston, Massachusetts, United States of America; 2 Broad Institute, Cambridge, Massachusetts, United States of America; 3 Children's Hospital Boston, Boston, Massachusetts, United States of America; 4 HUNT Research Centre, Department of Public Health and General Practice, Norwegian University of Science and Technology, Levanger, Norway; 5 St. Olav Hospital, Trondheim University Hospital, Trondheim, Norway; 6 Department of Public Health and General Practice, Norwegian University of Science and Technology, Trondheim, Norway; 7 National Institute for Health and Welfare, Helsinki, Finland; 8 Department of Laboratory Medicine, Children's and Women's Health, Norwegian University of Science and Technology, Trondheim, Norway; 9 Institute for Molecular Medicine Finland (FIMM), University of Helsinki, Helsinki, Finland; 10 Department of Internal Medicine, Division of Cardiovascular Medicine, University of Michigan, Ann Arbor, Michigan, United States of America; 11 Estonian Genome Project, University of Tartu, Tartu, Estonia; 12 Wellcome Trust Sanger Institute, Wellcome Trust Genome Campus, Hinxton, United Kingdom; 13 Department of Medical Genetics, University of Helsinki and University Central Hospital, Helsinki, Finland; 14 Genetics of Complex Traits, Peninsula Medical School, University of Exeter, Exeter, United Kingdom; Georgia Institute of Technology, United States of America

## Abstract

Common genetic variants have been shown to explain a fraction of the inherited variation for many common diseases and quantitative traits, including height, a classic polygenic trait. The extent to which common variation determines the phenotype of highly heritable traits such as height is uncertain, as is the extent to which common variation is relevant to individuals with more extreme phenotypes. To address these questions, we studied 1,214 individuals from the top and bottom extremes of the height distribution (tallest and shortest ∼1.5%), drawn from ∼78,000 individuals from the HUNT and FINRISK cohorts. We found that common variants still influence height at the extremes of the distribution: common variants (49/141) were nominally associated with height in the expected direction more often than is expected by chance (*p*<5×10^−28^), and the odds ratios in the extreme samples were consistent with the effects estimated previously in population-based data. To examine more closely whether the common variants have the expected effects, we calculated a weighted allele score (*WAS*), which is a weighted prediction of height for each individual based on the previously estimated effect sizes of the common variants in the overall population. The average *WAS* is consistent with expectation in the tall individuals, but was not as extreme as expected in the shortest individuals (*p*<0.006), indicating that some of the short stature is explained by factors other than common genetic variation. The discrepancy was more pronounced (*p*<10^−6^) in the most extreme individuals (height<0.25 percentile). The results at the extreme short tails are consistent with a large number of models incorporating either rare genetic non-additive or rare non-genetic factors that decrease height. We conclude that common genetic variants are associated with height at the extremes as well as across the population, but that additional factors become more prominent at the shorter extreme.

## Author Summary

Although there are many loci in the human genome that have been discovered to be significantly associated with height, it is unclear if these loci have similar effects in extremely tall and short individuals. Here, we examine hundreds of extremely tall and short individuals in two population-based cohorts to see if these known height determining loci are as predictive as expected in these individuals. We found that these loci are generally as predictive of height as expected in these individuals but that they begin to be less predictive in the most extremely short individuals. We showed that this result is consistent with models that not only include the common variants but also multiple low frequency genetic variants that substantially decrease height. However, this result is also consistent with non-additive genetic effects or rare non-genetic factors that substantially decrease height. This finding suggests the possibility of a major role of low frequency variants, particularly in individuals with extreme phenotypes, and has implications on whole-genome or whole-exome sequencing efforts to discover rare genetic variation associated with complex traits.

## Introduction

Height is a highly heritable trait, with estimates of heritability as high as 90% [1]. Recent genome-wide association studies of height have discovered over 180 common variants associated with height [2]. These variants have small effect sizes and collectively explain approximately 10% of the heritability. While these 180 common variants are robustly associated with height when studied as a quantitative trait in the general population, it is not known whether these variants have similar associations with stature in individuals at the extreme tails of the height distribution. If these common variants do not show the expected association with stature at the extremes (based on their continuous distribution effect sizes), then other factors beyond common variants must contribute to extreme stature. Although there are multiple possible scenarios, one possible explanation is the existence of rare or low frequency variants with larger effect sizes, which have been proposed to explain a portion of the heritability not accounted for by the known common variants [3]–[5] and which may provide novel biological insights into mechanisms that affects height. Understanding the role of common variants in the tails of the height distribution will also provide methodological insight into the utility of extreme tails analysis for future genetic studies of quantitative traits.

In this paper, we describe our approach to determine whether common alleles known to be associated with height in the general population have the expected distribution in individuals from the extremes of the height distribution. We used DNA samples from individuals with extreme heights from two population-based cohorts of Finnish (FINRISK) and Norwegian (HUNT) ancestry and genotyped them for common variants known to be associated with height. Under a polygenic model in which there are many variants and each variant additively contributes a small effect to the phenotype, we found that for individuals within ∼2.81 standard deviations of the mean, the common variants have the predicted associations with height, consistent with their effect sizes estimated from the previous population study [2]. However, in individuals with more extreme short stature (the shortest 0.25% of the distribution), common variants play a less prominent role in explaining phenotype, and the data are consistent with various models in which rare variants, non-additive effects or rare non-genetic factors contribute to short stature in these individuals.

## Results

### Individual common variants are associated with height in the extremes

We attempted to genotype SNPs at the 180 loci previously associated with height in individuals from the short and tall extremes of the FINRISK and HUNT cohorts and then performed association analyses for each SNP with height using the Cochran-Mantel-Haenszel test and logistic regression respectively. In FINRISK, SNPs at 158 of the height loci were successfully genotyped in 181 short and 192 tall individuals from the 1% tails of the height distribution. In the HUNT study, SNPs at 160 of the height loci were successfully genotyped in 385 short and 456 tall individuals from the ∼1.5% tails of height. Here we focus on the 279 short and 309 tall individuals from the 1% tails of the HUNT study, so as to provide consistency with the FINRISK study. In both cohorts, the majority of SNPs had effect directions consistent with the published results [2] (HUNT 137/160, *p*<0.0001; FINRISK 122/155, *p*<0.0001) and there was a significant enrichment in SNPs reaching nominal significance for association with height (Table S1; Table S2). We then combined the data from both cohorts in a meta-analysis of 141 overlapping loci (Table S3). Ninety-one percent of SNPs (128/141, *p*<0.0001) had directions of effect consistent with previously published results [2] and 49 SNPs had p-values<0.05, as opposed to 7 expected by chance (*p*<5×10^−28^). This result confirms that, as a group, SNPs found to be associated with height in the general population are also associated with height at the extremes of the phenotypic spectrum.

### The effect sizes of individual common variants on height are similar in the extremes and the general population

We next tested whether the observed odds ratios (OR) are consistent with the expected odds ratios, based on the previously estimated effect sizes from the GIANT study [2] and study specific allele frequencies (see Materials and Methods). Overall, the number of SNPs with observed odds ratio greater than expected odds ratios was no different than expectation under the model of equal effect sizes in extremes and the general population (HUNT 79/160 SNPs, *p* = 0.94; FINRISK 75/155 SNPs, *p* = 0.48 and combined 75/141, *p* = 0.45); (Table S1; Table S2 and Table S3). Next, for each SNP we tested for a difference between the expected and observed odds ratio in the individual studies and in the meta-analysis. Overall there were no more or fewer significant associations than would be expected under the equal effect size model (Figure S1). This result demonstrates that the individual SNPs have similar effects at the extremes as in the general population.

### Weighted Allele Score (*WAS*) analysis: The additive effect of the common variants differs significantly from expected in the short extremes

**Figure 1:**
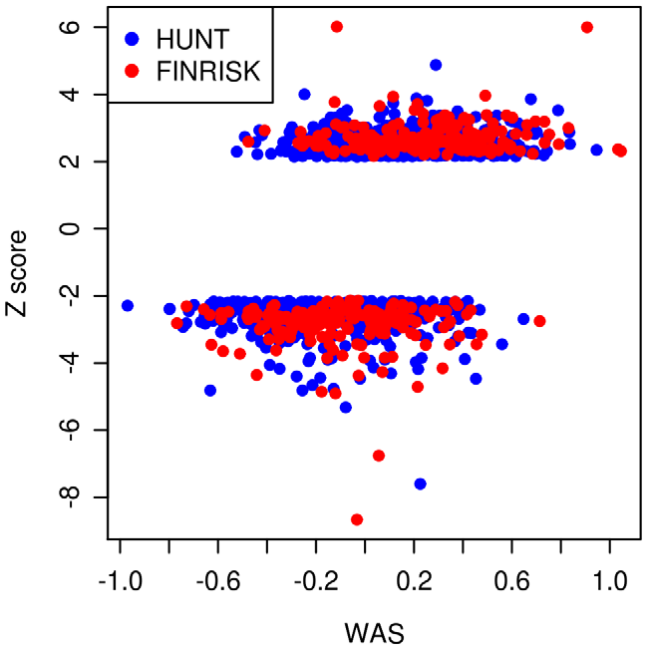
Plot of weighted allele scores (*WAS*) against Height Z-scores for HUNT and FINRISK cohorts. The plot shows the *WAS*, a measure of the genetic prediction of height by known common variants, against the height Z-scores. The tall individuals (Z-score>2.14) have generally larger *WAS* than the short individuals (Z-score<−2.14). Individuals from the HUNT study are labeled blue and individuals from the FINRISK study are labeled red.

After determining that the individual SNPs have similar effects at the extremes of the height distribution as in the general population, we then performed additional analyses on the combined set of height-associated variants. We asked whether extremely short and extremely tall individuals show overall enrichment of height-decreasing and height-increasing alleles, respectively, to the extent expected under a purely polygenic additive model. If the enrichment is less than expected, this result would suggest that the common variants are not explaining as much of the phenotypic variation in the extremes as in the general population. To test this possibility, we first calculated the weighted allele score (*WAS*) for each individual using the height-associated SNPs previously described. The *WAS* is the cumulative effect of all of the SNPs on height weighted by each SNP's estimated effect size (β). In Figure 1, we show a plot of each individual's *WAS* based on the 143 loci genotyped in both cohorts versus the individual height Z-scores. As expected, the *WAS* are significantly different between the tall extremes and the short extremes (*p*<3×10^−86^), with individuals in the tall extreme having higher *WAS* on average than individuals in the short extremes.

**Figure 2:**
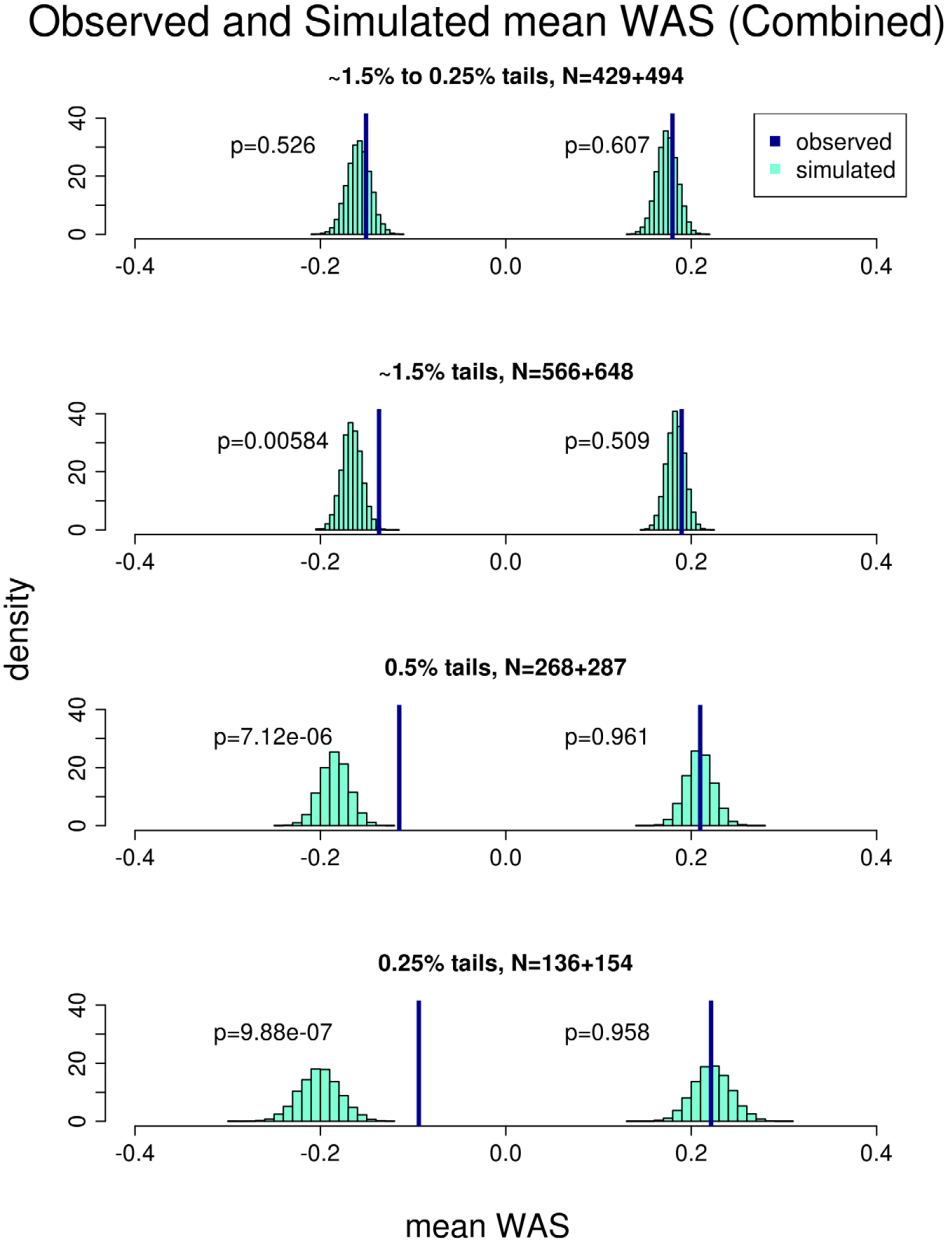
Comparison of the observed versus simulated mean weighted allele score (*WAS*) in the combined cohort. The plot shows the result of comparing the mean *WAS* of the short and tall individuals observed from both the HUNT and FINRISK cohorts against that obtained from simulation. Each row represents a different stratification of the extremes. The percentiles and number of individuals in the short and tall extreme respectively are listed for each stratum. The p-values represent the comparison between the observed and simulated mean *WAS*. The observed mean *WAS* for the tall individuals are not different from the simulation in any of the strata. The observed mean *WAS* for the short individuals is not different from the simulation in the first stratum. As a progressively more extreme sample is used, the short individuals' mean *WAS* becomes progressively more significantly different than the simulation.

We then tested whether the *WAS* in the short and tall groups are within expectations based on the population specific allele frequencies and previously estimated effect sizes of these SNPs, assuming a purely polygenic model. To generate the distribution of *WAS* under these expectations, we simulated populations that mimicked our ascertainment of extreme samples from the HUNT and FINRISK populations (see Materials and Methods). For each cohort, we compared the observed mean *WAS* with the distribution of mean *WAS* under the simulated model (Figure S2 and Figure S3). For the HUNT study the sample of 1224 individuals from the middle of the distribution suggest our modeling is behaving as expected (Figure S2). Finally, we analyzed the data by combining both studies using the 143 SNPs present in both data-sets (Figure 2). In each study separately and in the combined analysis, the mean observed *WAS* for the tall individuals was within expectation, but we observed a significant upward deviation of the mean observed *WAS* in the short extremes (*p* = 0.006 for the combined-analysis). These results suggest that the collective effect of the common variants in the short extremes do not account for as much of the phenotypic variation in height as predicted from the effects seen in the general population.

### The reduced effect of common variants is limited to the most extreme short individuals

Having established that the common variants do not explain as much phenotypic variation in the short extremes, we then sought to determine if this finding was accentuated in individuals with the most extreme short stature. We stratified our analysis in several ways (Figure 2; Figure S2; Figure S3). First, we removed the most extreme individuals: those below the 0.25 percentile and above the 99.75 percentile. In the combined cohorts, the mean observed *WAS* in the short extremes was no longer significantly different than expected (*p* = 0.526), indicating that the shift in *WAS* is driven by the most extremely short individuals. To further explore this hypothesis, we then selected more extreme individuals at two thresholds, including only the top and bottom 0.5% or 0.25% of the population (See Materials and Methods). For both strata, there was a more pronounced deviation of the mean observed *WAS* in the short extremes (*p* = 7.12×10^−6^ and *p* = 9.88×10^−7^ for the 0.5% and 0.25% extremes respectively), but again no deviation in the tall extremes. Similar observations occurred when we analyzed the cohorts separately using the same stratification procedure (Figure S2; Figure S3). We repeated the analysis using Z-scores based on inverse normal transformation, and with the three −6 SD outliers removed, and the results were essentially unchanged. The difference observed in the *WAS* analysis is also supported by the individual SNP analysis: when we performed the combined analysis described above for the 0.25% extremes rather than the entire cohort, 60% (84/139) of the SNPS have an observed effect size smaller than expected (*p* = 0.02) (Table S4). This analyses clearly suggest that the initial marginally significant shift of the mean observed *WAS* in the short extremes is primarily driven by the most extreme short individuals. Therefore, in general, as one selects individuals with more extreme short stature, in particular those with heights below the 0.25 percentile, the common variants play a much smaller role in explaining stature, indicating that there must be other factors contributing to the phenotypic variation in these extremely short individuals.

### Low frequency or rare variants with larger effect sizes could explain the phenotypic variation in the short extremes

**Figure 3:**
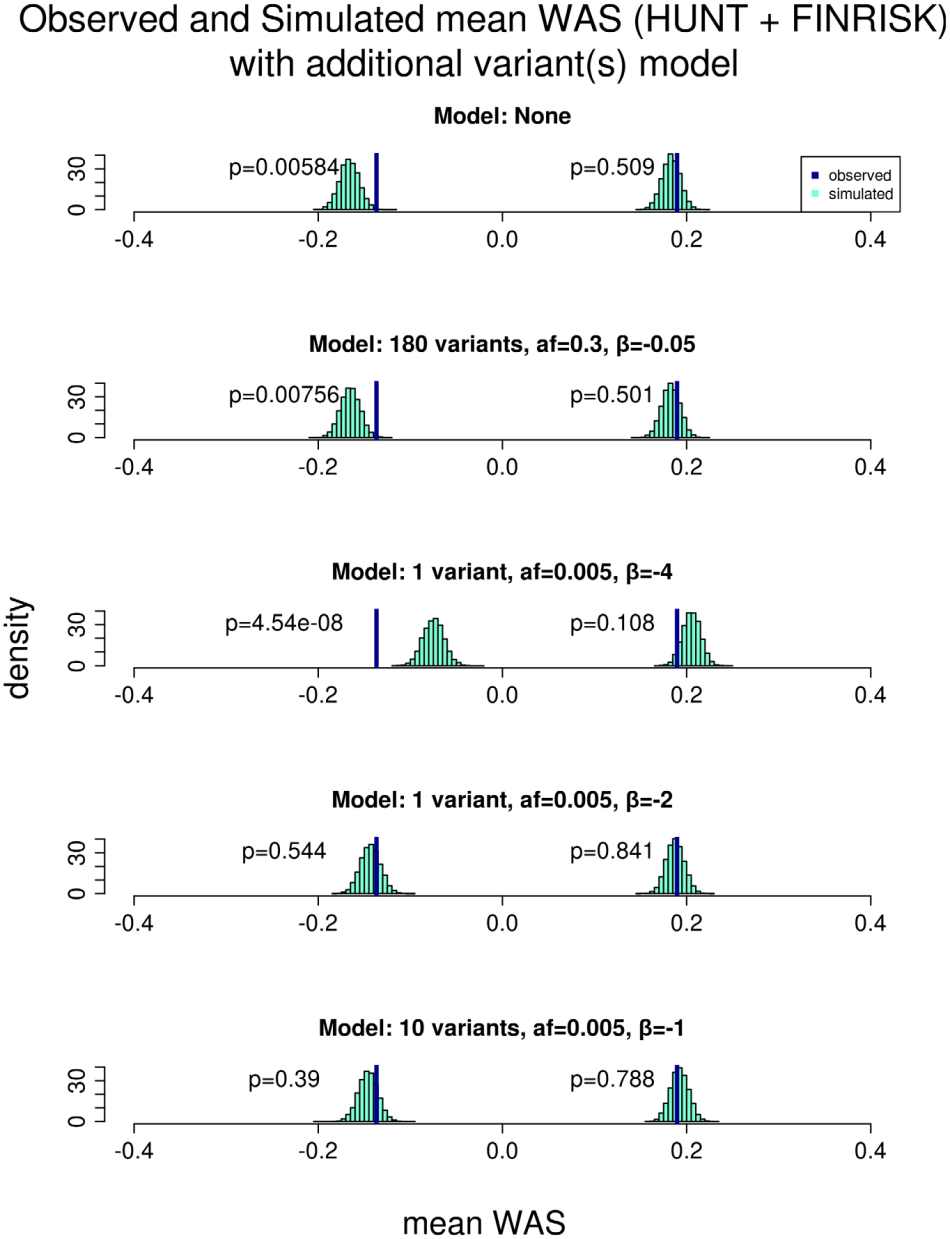
Comparison of the observed versus simulated mean *WAS* with models incorporating additional variants. The plot shows the result of comparing the mean *WAS* of the short and tall individuals observed from both the HUNT and FINRISK cohorts against that obtained from simulation with different scenarios of additional variants. All rows use the approximate 1.5% tails of the height distribution as extremes, resulting in 566 short and 648 tall individuals. The 1^st^ row shows the result where the model has no additional variants affecting height and thus is identical to that from the 2^nd^ row of Figure 2. The 2^nd^ row shows a model where there are 180 additional common variants that slightly decreases height (allele frequency = 0.3 and effect size (β) = −0.05). This model does not result in any significant change to the simulated *WAS* of the short individuals and the observed *WAS* is still significantly different (*p* = 0.00756). The 3^rd^ row shows a model where there is 1 additional low frequency variant with a large height decreasing effect (allele frequency = 0.005 and effect size (β) = −4). This model results in a large shift in the simulated *WAS* of the short individuals to the right. The observed *WAS* is still significantly different (*p* = 4.54×10^−8^) than the simulation but in the opposite direction and thus is not consistent with our data. The 4^th^ row shows a model where there is 1 additional low frequency variant that decreases height significantly (allele frequency = 0.005 and effect size (β) = −2). This model results in a shift in the simulated *WAS* of the short individuals to the right such that the observed *WAS* is no longer different from the simulation (*p* = 0.544). The 5^th^ row shows a model where there are 10 additional low frequency variants that moderately decreases height (allele frequency = 0.005 and effect size (β) = −1). This model also results in a shift in the simulated *WAS* of the short individuals to the right such that the observed WAS is no longer different from the simulation (p = 0.39). The final two models are consistent with our observed data.

We hypothesized that lower frequency and rare genetic variants with larger effect sizes than the common variants may explain the phenotypic variation in the short extremes. To test this hypothesis, we performed population simulations with rare-variants of various allele frequencies and effect sizes, and asked if our observed data were consistent with these simulated scenarios (Figure 3; Figure S4; Figure S5). As a negative control, we first modeled an additional 180 SNPs, each with allele frequency of 0.3 and average effect sizes of −0.05 SD, which is similar to the allele frequency and effect size for previously discovered common variants associated with height. In this simulation, the mean *WAS* distribution did not change, indicating that adding additional common variants of similar effect sizes cannot explain the phenotypic variation in the short extremes. We then modeled a single rare variant of very large effect: frequency 0.005 and effect size of −4 SD. In this model, the mean *WAS* distribution in the extremely short individuals shifts more than we observed in our population. This simulation essentially excludes the possibility of a 0.5% variant of very large effect within our cohort. Such a variant would also be likely to be discovered in linkage studies of several thousand sib-pairs [6].

However, there are several rare variant models that would likely not have been detected in previous linkage analyses of height and generate a shift in the mean *WAS* consistent with our observed data (Figure 3; Figure S4; Figure S5). One such possibility is a single low frequency variant (allele frequency = 0.005) with an effect size of −2 SD; another model consistent with our data includes 10 variants each with an allele frequency of 0.005 and a moderate effect size of −1 SD. These simulations suggest that individuals with very short stature may harbor small numbers of low frequency variants of moderately large effect or a greater number of low frequency variants of moderate effects contributing to their short stature. This result stands in contrast to the remainder of the height distribution in which a polygenic effect of common and rare variants with small effects could explain the majority of the heritability of height, even though only a small percentage of height-associated common variants have been identified.

### Sibling analysis provides support for a different genetic architecture in extreme short individuals

To provide further support for a different genetic architecture in individuals in the extreme short tails we performed an analysis in siblings from the HUNT study. We queried the entire HUNT database (N = 106,455) and identified 21,365 siblings pairs. The correlation of age and gender adjusted height between siblings was high (r = 0.466). We then identified 98 individuals (aged between 20–70 yrs) with a Z-score<−2.81 (∼0.25% tails) and 80 with a Z-score>2.81 who also had at least one sibling in the database (the results are similar if we use inverse normal transformation). The average height Z-score for the siblings of the extreme short group was −0.97 (95% CI: −0.80, −1.15); the average Z-score for the full siblings of the extreme tall group was 1.29 (95% CI: 1.14, 1.45) which are significantly different (t-test, *p* = 0.007 after reversing signs for the short group). We then performed this same analysis for the 0.25% to 1.5% tails individuals and there was no significant difference in z-scores of siblings between the short (−1.05 95% CI: −1.13, −0.97) and tall (1.11 95% CI: 1.03, 1.18) groups (t-test, *p* = 0.28). So the differential regression to the mean appears to be limited to the shortest ∼0.25% of individuals with this group regressing more quickly than the tall extreme group. This is consistent with the results we observe with the weighted allele score (*WAS*) approach.

We do not have the twin data that would allow us to separate out the environmental and genetic effects in this group and our data is consistent with both. If the effect were due to genetics, then a model with *de novo* mutations and/or multiple recessive rare variants could cause an increased regression to the mean in extremely short individuals, although there are other plausible explanations.

## Discussion

We have assessed whether common variants robustly associated with height in the general population also associate with height at the extreme tails of the height distribution. We further tested whether this association is to the extent expected under a purely polygenic model. By genotyping ∼160 height SNPs identified from the GIANT study [2] (that explain ∼10% of the population variation in height) in individuals from the ∼1% tails of height from two large population based cohorts, we have shown that the polygenic model can explain the associations in the ∼1% tails of height. However, our data indicate that the polygenic model starts to break down in extreme short individuals near the 0.25 percentile cut off. This conclusion is supported by our sibling analysis, which demonstrated that siblings in the 0.25% short tail regress to the mean more than those in the 0.25% tall group. Interestingly, the overall height distribution also shows a slight asymmetric deviation from normality, with an excess of individuals with extremely short stature but not for extremely tall stature.

While in general the individuals in the ∼1% tails carry as many height increasing alleles as would be predicted based on their height, there was a clear deviation for individuals in the shortest 0.25% tail. On average, these individuals carry significantly more “tall” alleles at the 160 SNPs than would be expected if common alleles were explaining their short stature. This suggests that the heights of these individuals are explained by factors other than common variants. Our simulations suggest that rare variants could explain this difference in the 0.25% shortest tail. For example, 10 rare variants with modest effects on height (1SD) are consistent with our observed data, as is a single variant with a 2SD effect. The sibling analysis also suggests a role for *de novo* or multiple recessive variants in the extreme short individuals. While rare height-decreasing variants of large effect are a plausible explanation, there are many other genetic models consistent with our data, including a mixture of height-decreasing with a smaller number of height-increasing rare variants, or variants having non-additive effects. While non-additive genetic effects could explain the data, no evidence was found for dominance or gene-gene interaction effects for the SNPs used in this study in the original GIANT publication [2]. It is also possible that these individuals are short for non-genetic reasons. One could suggest that these individuals are short because of differences in ancestry, but we have taken steps to remove any possible ethnic outliers from our extremes (See Materials and Methods, Figure S6 and Figure S7). Measurement or recording error is another possibility, although the fact that the tall group does not show this effect (which presumably is equally likely to contain measurement error as the short group) suggests this is an unlikely explanation. Non-genetic factors could also be a possibility, for example, poor early-life nutrition, severe infection, or other chronic childhood diseases could have prevented these individuals from reaching their genetic height potential.

This result also suggests that these families would be good candidates to investigate in sequencing studies, as they may be enriched for rare or de novo, higher penetrance alleles. More generally, the weighted allele score (*WAS*) method developed here could be used to select individuals to sequence in the search for these types of rarer variants, not only for height but also for other polygenic traits and diseases. Specifically, individuals in the extreme tails of a trait distribution who have an unexpectedly high or low weighted allele score may be particularly useful to sequence, especially if multiple relatives with these characteristics were present in the extreme tails.

Our study also demonstrates empirically that selecting individuals from the extreme tails of a complex trait distribution is an efficient approach for genetic studies, as was proposed both for linkage studies [7], [8] and association studies [9], [10]. Despite a quite modest sample size (N<1000), we replicated a large fraction of the individual SNPs identified in the GIANT study in our extreme height analysis. Ninety-one percent of the SNPs had odds ratios that were directionally consistent with the direction in the published GIANT study (*p*<0.0001), and 35% (49/141) of SNPS had *p*<0.05 in the consistent direction. Our analyses also demonstrate that, outside of the 0.25% tails, this level of association is entirely consistent with that expected given the extreme tail ascertainment of our samples and the individual SNP continuous distribution effect sizes. Given this result, the ascertainment of our 923 samples from the ∼1% to 0.25% tails provides equivalent power to approximately 6000 samples randomly selected from the general population for a variant explaining approximately 0.1% of the variation in height. Indeed, the ability to detect associations in samples ascertained for extreme phenotypes has been recently demonstrated in studies of bone mineral density [11], body mass index [12], triglyceride levels [13], and type 2 diabetes (using a liability threshold model [14]). Also, our results suggest that the statistical power of detecting these small effect variants would be reduced if we were to include the most extreme tails of the phenotypic distribution (in our case, the shortest 0.25% of individuals), consistent with predictions made based on simulation studies of mixtures of common and rare variants [15]. Nonetheless, our findings suggest that the use of individuals with the most extreme phenotypes could be particularly valuable to detect rarer variants with larger effect sizes more efficiently.

In conclusion, we have shown that common genetic variants associated with height in the general population are also associated with height at the ∼1% tails of the height distribution. Our data suggest that common variants play less of a role, and the effect of rarer larger-effect alleles and/or strong environmental factors start to predominate around the 0.25% extreme. This finding may also have broader implications for studies of disease, in that the polygenic model may apply well to those diseases that represent the tails of an underlying normal distribution, but perhaps less well to diseases that correspond to more extreme phenotypes.

## Materials and Methods

### Ethics statement

Both studies were conducted according to the principles expressed in the Declaration of Helsinki. Attendance was voluntary, and each participant signed a written informed consent including information on genetic analyses. Local institutional review boards approved study protocols.

### Subjects

#### The HUNT study

The Nord-Trøndelag Health Study (HUNT) is a comprehensive population based health study (www.ntnu.edu/hunt) with personal and family medical histories on approximately 120,000 people from Nord-Trøndelag County, Norway, collected during three intensive studies (HUNT 1, 2, and 3). Inviting all citizens aged 20 and over, information was collected from self-reported questionnaires consisting of >200 health-related questions, standardized clinical examinations, urine and non-fasting venous blood sample. The population in Nord-Trøndelag County is ethnically homogeneous, <3% of non-Caucasian ethnicity, making it especially suitable for epidemiological genetic research. Height was measured by trained personnel to the nearest 1.0 cm with the participants wearing light clothes without shoes according to standardized methods [16].

For this study we sourced data from HUNT 2 (1995–97) in which 65,258 individuals participated (71.2% of invited). We generated age and gender standardized height for the whole population, and selected the shortest 1000 individuals and the tallest 1000 individuals from the 54,909 participants aged between 18 and 70 yrs. We removed known 1st degree relatives based on information from the Medical Birth Registry of Norway, those reporting to be living outside of Norway their first year of life, and those with low DNA concentrations. We then genotyped the remaining shortest 471 individuals (<−2.14 SDs) and the tallest 479 individuals (>2.14 SDs) from the cohort. Mean height and age of the extreme tails are given in Table S5. We also genotyped 1,458 individuals of all ages with a Z-score between +/−2 SDs as our middle group.

#### The FINRISK Study

FINRISK is a Finnish national survey on risk factors of chronic and non-communicable diseases. It is carried out every five years since 1972 using independent, random and representative population samples from different parts of Finland [17]. For this study, we selected individuals from 4 different sub-populations divided by geography (East vs. West Finland) and gender (Table S6). Individuals aged 25 to 74 years were included. We then took approximately the tallest and shortest 50 individuals (Table S6) from each tail of the distribution from each sub-population (extremes) and performed genotyping.

### Genotyping and quality control

#### The HUNT study

Blood sampling was done whenever subjects attended HUNT 2. DNA was extracted from peripheral blood leukocytes from whole blood or blood clots stored in the HUNT Biobank, using the Puregene kit (Gentra Systems, Minneapolis, MN) manually or with an Autopure LS (Gentra Systems). Laboratory technicians were blinded to the results of the height measurements. Details on the DNA extraction and the HUNT Biobank are described elsewhere [16].

Genotyping of short and tall individuals were done at the Norwegian University of Science and Techonology, Norway using the iSelect Metabochip (Illumina, San Diego, CA) and the Infinium HD ultra protocol. Each 96-well plate included both tall and short individuals and one sample of identical reference DNA. Genotype calling was done using GenTrain version 2.0 in GenomeStudio V2010.3 (Illumina, San Diego, CA). Genotyping of the middle group was done on the Metabochip at the Center for Inherited Disease Research (CIDR, MD) and called with BeadStudio 3.3.7 with Gentrain version 1.0 (Illumina, San Diego, CA).

Samples that did not meet a 99% completion threshold were excluded from further analysis (N = 19; 0.7%). Additional post-genotyping exclusions based on gender discrepancy (N = 11) and first-degree relatedness (pi-hat >0.2; N = 152, 6.3%) were done using PLINK [18]. Ethnic outliers (N = 174, 7.2%) were excluded using the EIGENSTRAT software package [19]. After quality assessment 2,063 individuals (85.7%) remained for further analysis, 385 (81.2%) short, 456 (95.2%) tall and 1,224 (83.9%) individuals in the middle group.

106 SNPs of the 180 GIANT height hits were directly typed on the Metabochip. In addition, we used the SNP Annotation and Proxy Search to map 54 of the remaining 74 SNPs with a HapMap r2>0.8 linkage disequilibrium proxy result [20]. These 160 SNPs (i.e. 106 directly typed and 54 proxies) were used in subsequent analyses. All SNPs showed a genotyping success rate >98% and were in Hardy Weinberg equilibrium.

#### The FINRISK study

We directly genotyped the samples for the 180 previously identified height SNPs. The genotyping was done at Children's Hospital Boston using Sequenom iPLEX genotyping (Sequenom, Inc, San Diego, CA, USA). In total, 186 short individuals and 192 tall individuals were successfully genotyped for 158 SNPs. All 158 SNPs had a genotyping success rate ≥90% and the overall genotyping rate was 97.85% (Table S7). One of these SNPs (rs1809889) is not part of the 180 GIANT SNPs, but data were available for this SNP from the GIANT meta-analysis so it was included in our analysis.

We genotyped an additional 49 ancestry informative markers (AIMs) to identify ethnic outliers [21]. We inputted genotype data from our subjects as well as the reference HAPMAP samples (CEU, YRI, CHB+JPT) for the 49 AIMs together with 130 height SNPs into Structure 2.3.3 [22]. We detected 5 ethnic outliers with >10% Asian ancestry who were excluded from further analysis leaving a total of 181 short and 192 tall individuals as our FINRISK study group.

### Statistical analysis

#### Individual SNP analysis

For FINRISK, we calculated the observed odds ratio for each of our 158 SNPs using the Cochran-Manzel-Hansel test, which is a stratified chi-square test. We stratified the individuals into 4 sub-cohorts based on geography and gender (Table S6) and performed the test using PLINK [18]. The observed odds-ratio for each SNP was recorded, along with the 95% confidence interval. For HUNT the observed odds-ratio and 95% confidence intervals and the single association analysis was performed using logistic regression in PLINK.

For both cohorts, we calculated the expected odds ratio for each SNP by estimating the odds of the height-increasing versus the height-decreasing allele in both the tall extremes (cases) and the short extremes (controls) assuming a standard normal distribution for standardized height, i.e. height∼*Normal(0,1)*. For a given SNP, we defined the height-increasing effect size as *β* and the height-increasing allele frequency as *p*. The mean height for the height-increasing allele would be *M_i_* = *β p* and the mean height for the height-decreasing allele would be *M_d_* = −*β* (1−*p*). The variance of height for the both alleles would be *V* = 1−*β^2^ p (1−p)*. We then calculated the odds of observing the height-increasing allele versus the height-decreasing allele for both the tall extremes (cases) and the short extremes (controls) by taking the ratio of the probabilities of each allele being seen in the cases and the controls respectively. These are calculated as:
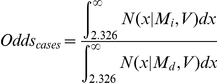


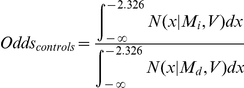
where *N(x|M,V)* denotes the density function at *x* of a Normal distribution with mean *M* and variance *V*. We use a cut-off of +/−2.326 to denote the approximate 1% tails. We then calculated the expected odds-ratio by taking the ratio between *Odds_cases_* over *Odds_controls_*, i.e.
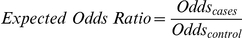
To assess whether individual SNPs had odds ratios significantly different from expectation, we generated upper and lower 95% confidence limits for the expected distribution based on the GIANT beta and standard errors estimates as above, and used the natural log of these confidence limits to estimate an approximate standard error for the expected odds ratio, i.e.
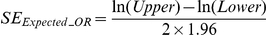
We then assessed significance by a Z-test of the difference between observed odds ratio and expected odds ratio to obtain the Zscore, i.e.
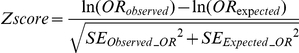



#### Meta-analysis

The HUNT and FINRISK studies genotyped different sets of SNPS, with only 98 of the SNPs matching exactly across the studies. We therefore used forty-three of the HUNT SNPs that had r^2^>0.8 HapMap proxies with a genotyped FINRISK SNPs (Table S3). We used the inverse variance method to meta-analyze the odds ratios for these 141 SNPs from the two studies. As opposed to the individual studies, where study specific allele frequencies were used, we used the GIANT allele frequency information to generate the expected odds ratios for the meta-analysis. This did not appreciably affect the results for individual SNP analysis within the individual studies, and the meta-analyzed results were consistent to those in the two individual studies.

#### Modeling the Weighted Allele Score (WAS)

To calculate the Weighted Allele Score (*WAS*) for each individual, we took the sum of the effective allele dosages of the height SNPs multiplied by their respective estimated effect sizes (βs) using the Stage 1 betas from the GIANT study, as shown in the formula below.
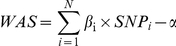

*β* and *SNP* are the effect size and effective allele dosage (0, 1 or 2) of the height SNPs and *WAS* is the weighted allele score. *N* is the total number of SNPs available to calculate the weighted allele score. α is the mean of the sum such that the expected *WAS* is 0 as shown by the formula below.
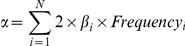

*Frequency* is the allele frequency of the effect allele obtained from the Finnish or HUNT estimates.

We calculated the statistical difference between the *WAS* of the short versus the tall individuals by performing a 2-tailed 2-sample t-test to obtain the respective p-value. All the calculations were done using the R statistical software package.

#### Obtaining Finnish allele frequency estimates

The allele frequency estimate for each SNP was obtained by taking only the Finnish individuals from the GIANT height study and calculating the expected allele frequency. The cohorts used were the FUSION NIDDM Case control study from Finland, the GenMets Case control study from Finland and the FINRISK component of the MIGen cohort. The total number of individuals used for obtaining the estimates is 3618.

#### Simulating the distribution of WAS under the null model

The null model assumes that the only factors determining height (Z-score) are the cumulative additive effects of the GIANT height SNPs and noise. We modeled the Z-score with the formula below.
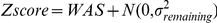

*Zscore* is the height Z-score, *N*(0, σ^2^_remaining_) is a normally distributed random variable with mean 0 and variance σ^2^_remaining_. σ^2^_remaining_ is calculated such that the variance of *Zscore* is 1, i.e. σ^2^_remaining_ is 1−var(*WAS*). The variance of *WAS* can be calculated with the formula below,
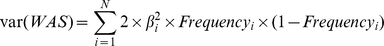
On the other hand, a simulated individual's effective allele dosage is obtained by sampling from a set of binomial distributions with *N* = 2 and *p* being the allele frequencies of each SNP. The simulated effective allele dosages can then be used to calculate each individual's *WAS*. The simulation approach for each cohort was modeled to mirror the methods of subject selection.

#### Simulating FINRISK

For the FINRISK study, the simulations were performed using the following steps. We first generated the effective allele dosages for each SNP for 200,000 individuals by random sampling. We then randomly sampled 4271, 6582, 5025 and 7610 individuals to represent the 4 sub-populations and obtained their Z-scores using the previously described modeling. For each subgroup, we picked the appropriate number of the most extreme individuals to mimic the actual sample selection. We then pooled the short and tall extremes together and randomly dropped individuals to obtain exactly 181 short extremes and 192 tall extremes. We then randomly drop SNPs from the simulated individuals to mimic the missing genotype rate in FINRISK and then calculate the Weighted Allele Score (*WAS*) for each simulated individual. This simulation process was repeated 10,000 times. For the stratified analyses of various height cut-offs, we adjusted the numbers of selected individuals in each strata by taking the floor of the expected number of individuals in that strata. In our cohort, the top 0.5% extremes included 21, 32, 25 and 38 individuals from each tail of the 4 sub-populations respectively, and for the top 0.25% extremes included 10, 16, 12 and 19 individuals from each tail of the 4 sub-populations. For the top ∼1% to 0.25% extremes, we included all our extremes but excluded the top 10, 16, 12 and 19 individuals from each tail of the 4 sub-populations.

#### Simulating HUNT

The simulations for HUNT were performed as follows. We generated the effective allele dosages for each SNP for 400,000 individuals by random sampling. We then randomly selected 50,000 individuals and obtained their Z-scores.

We then selected all short and tall extremes with a Z-score cut-off of −2.14 and +2.14 respectively. Next, we randomly selected 385 short extremes and 456 tall extremes and calculated the *WAS*. This process was repeated 10,000 times. As in the FINRISK simulation, the number of individuals varies for each stratified analysis. Because we performed stratified analyses for varying levels of height cut-offs, our definition for the top 0.5% extremes is a Z-score cut-off below −2.57 and above +2.57 and for the top 0.25% extremes is a Z-score cut-off below −2.81 and above +2.81. For the top ∼1.5% to 0.25% extremes, we used only extremes that had Z-scores between −2.14 and −2.81 for the short extremes and between 2.14 and 2.81 for the tall extremes.

#### Determining if the mean observed *WAS* is significantly different from the simulated expectation

We evaluated the significance of the mean observed *WAS* by determining the p-value of the mean observed *WAS* from the null distribution of the mean *WAS* obtained from the simulations. The two-tailed p-value is calculated by evaluating the mean observed *WAS* from Normal(μ_simulation_, σ^2^_simulation_) where μ_simulation_ is the mean of the mean *WAS* and σ^2^_simulation_ is the variance of the mean *WAS* from the simulations.

#### Modeling rare-variants with moderate to large effect sizes

Modeling the rare-variant effect into the simulation is accomplished by adding an additional rare-variant term into the calculation of the height Z-score without changing the definition of *WAS* as shown in the equation below.
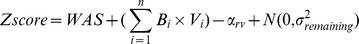
where *n* is the number of independent rare-variants, *B* represents the effect size of the rare-variants, and V is the allele dosage of the rare-variant. α_rv_ is the mean of the rare-variants score such that the rare-variants do not change the expected Z-score, i.e. the expected Z-score is still 0. Similarly, α_rv_ can be calculated by the following formula,
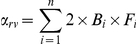
σ^2^_remaining_ in this case will have to be adjusted for the rare-variants such that the variance of the Z-score remains at 1, i.e. σ^2^_remaining_ is 1−var(*WAS*)−var(Σ *B V*). *F* is the allele frequency of the rare-variants. Simulations done with modeling rare-variants are identical to the prior simulations of FINRISK or HUNT except that the new terms are used for calculating the Z-score.

## Supporting Information

Figure S1 QQ Plot of p-values for individual SNPs based on the meta-analysis of HUNT and FINRISK. The figure shows a Q-Q plot of the p-values of the difference between the observed odd-ratios and the expected odd-ratios.
(TIF)

Figure S2 Comparison of the observed versus simulated mean weighted allele score (*WAS*) in the HUNT study. The plot shows the result of comparing the mean *WAS* of the short and tall individuals observed in the HUNT cohort against that obtained from simulation. Each row represents a different stratification of the extremes identical to those defined in Figure 2. The plot also show the mean *WAS* of 1224 non-extreme individuals taken from the middle of the height distribution. There is no difference between the mean *WAS* of the non-extreme individuals from that obtained from simulation (*p* = 0.56).
(TIF)

Figure S3 Comparison of the observed versus simulated mean weighted allele score (*WAS*) in the FINRISK study The plot shows the result of comparing the mean *WAS* of the short and tall individuals observed in the FINRISK cohort against that obtained from simulation. Each row represents a different stratification of the extremes identical to those defined in Figure 2.
(TIF)

Figure S4 Comparison of the observed versus simulated mean *WAS* by incorporating additional variants (HUNT only). The plot shows the result of comparing the mean *WAS* of the short and tall individuals observed from only the HUNT cohort against that obtained from simulation with different scenarios of additional variants. Each row represents a different scenario identical to those defined in Figure 3.
(TIF)

Figure S5 Comparison of the observed versus simulated mean *WAS* by incorporating additional variants (FINRISK only). The plot shows the result of comparing the mean *WAS* of the short and tall individuals observed from only the FINRISK cohort against that obtained from simulation with different scenarios of additional variants. Each row represents a different scenario identical to those defined in Figure 3.
(TIF)

Figure S6 Principal component plots from the HUNT study for the 0.25% short individuals versus all tall individuals. The first two principal components obtained from Eigenstrat analysis for the samples in the HUNT study are plotted. There is no significant difference in principal components between the short and tall groups.
(TIF)

Figure S7 Principal component plots from the HUNT study for the 0.25% short vs. the 0.25% tall individuals. The first two principal components obtained from Eigenstrat analysis for the samples in the HUNT study are plotted. There is no significant difference in principal components between the short and tall groups.
(TIF)

Table S1 Individual SNP analysis for HUNT cohort. The table shows the results for the SNPs used in the individual association analysis in the HUNT cohort.
(XLS)

Table S2 Individual SNP analysis for FINRISK cohort. The table shows the results for the SNPs used in the individual association analysis in the FINRISK cohort.
(XLS)

Table S3 Meta-analysis of individual SNPs for HUNT and FINRISK cohort. The table shows the results for the SNPs used in the meta-analysis of the HUNT and FINRISK cohorts.
(XLS)

Table S4 Meta-analysis of individual SNPs for HUNT and FINRISK cohort using only the top 0.25% tails. The table shows the results for the SNPs used in the meta-analysis of the HUNT and FINRISK cohorts using only the top 0.25% tails as extremes.
(XLS)

Table S5 Mean height and age of the individuals in the extreme tails of HUNT. Table of various stratification of extremes, the number of individuals in each strata separated by gender with their corresponding mean height (cm) and age (years) for the HUNT cohort.
(XLS)

Table S6 The FINRISK cohort divided into 4 sub-populations. The table shows the number of individuals used for each of the FINRISK sub-populations. The FINRISK cohort is sub-divided between male and female as well as individuals from east and west Finland.
(XLS)

Table S7 The genotyping rate of FINRISK. The table shows the number of SNPs genotyped in FINRISK and the genotyping rate for the short and tall extremes. The rate is calculated based on having 181 short individuals and 192 tall individuals.
(XLS)
