# Supplementary figures and images for: Common Variants Show Predicted Polygenic Effects on Height in the Tails of the Distribution, Except in Extremely Short Individuals
Source: PLoS Genet. 2011 Dec 29;7(12):e1002439. doi: 10.1371/journal.pgen.1002439 (PMC3248463; doi:10.1371/journal.pgen.1002439)

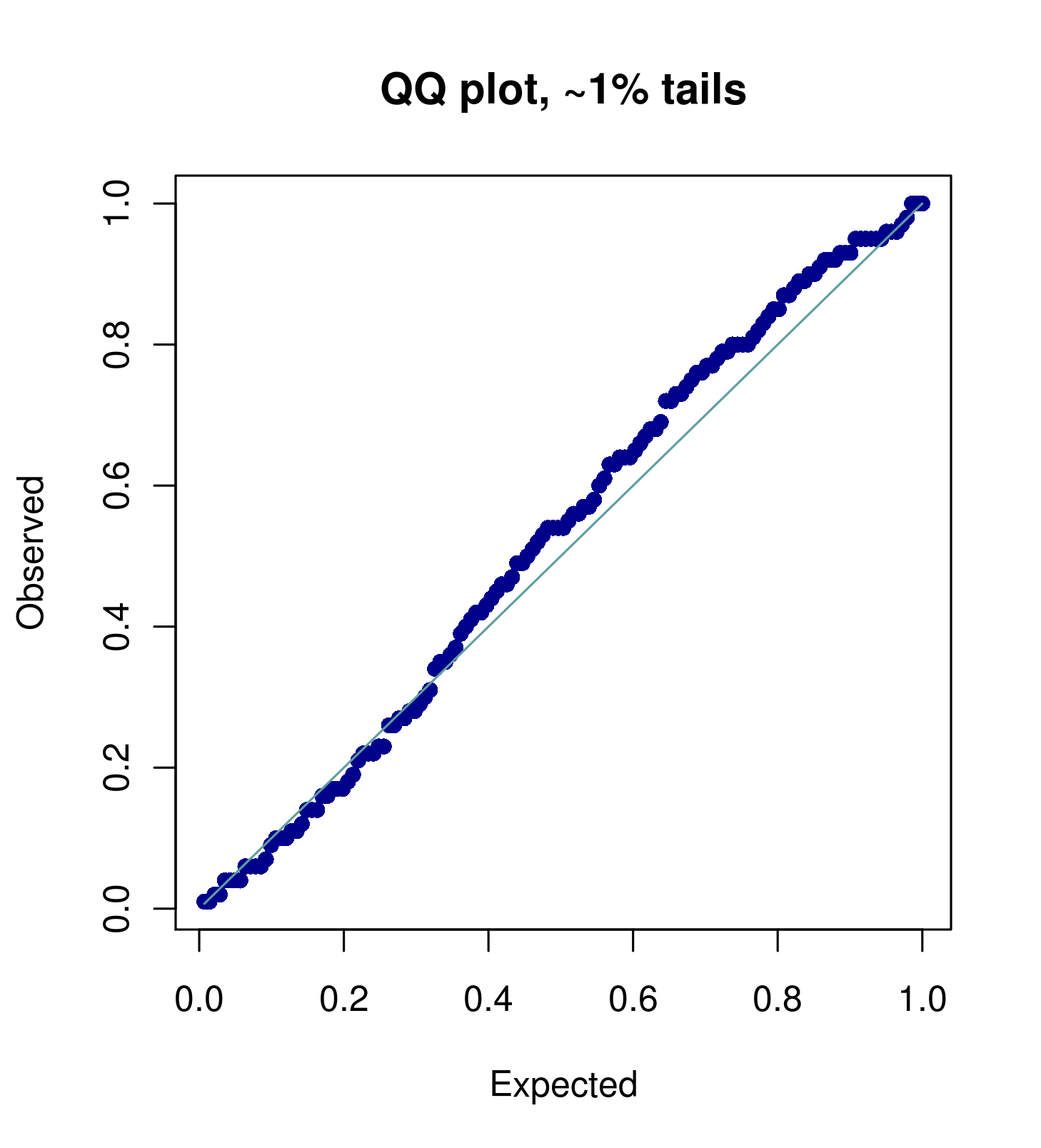

Supplement: Figure S1 — QQ Plot of p-values for individual SNPs based on the meta-analysis of HUNT and FINRISK. The figure shows a Q-Q plot of the p-values of the difference between the observed odd-ratios and the expected odd-ratios. (TIF) [file pgen.1002439.s001.tif]

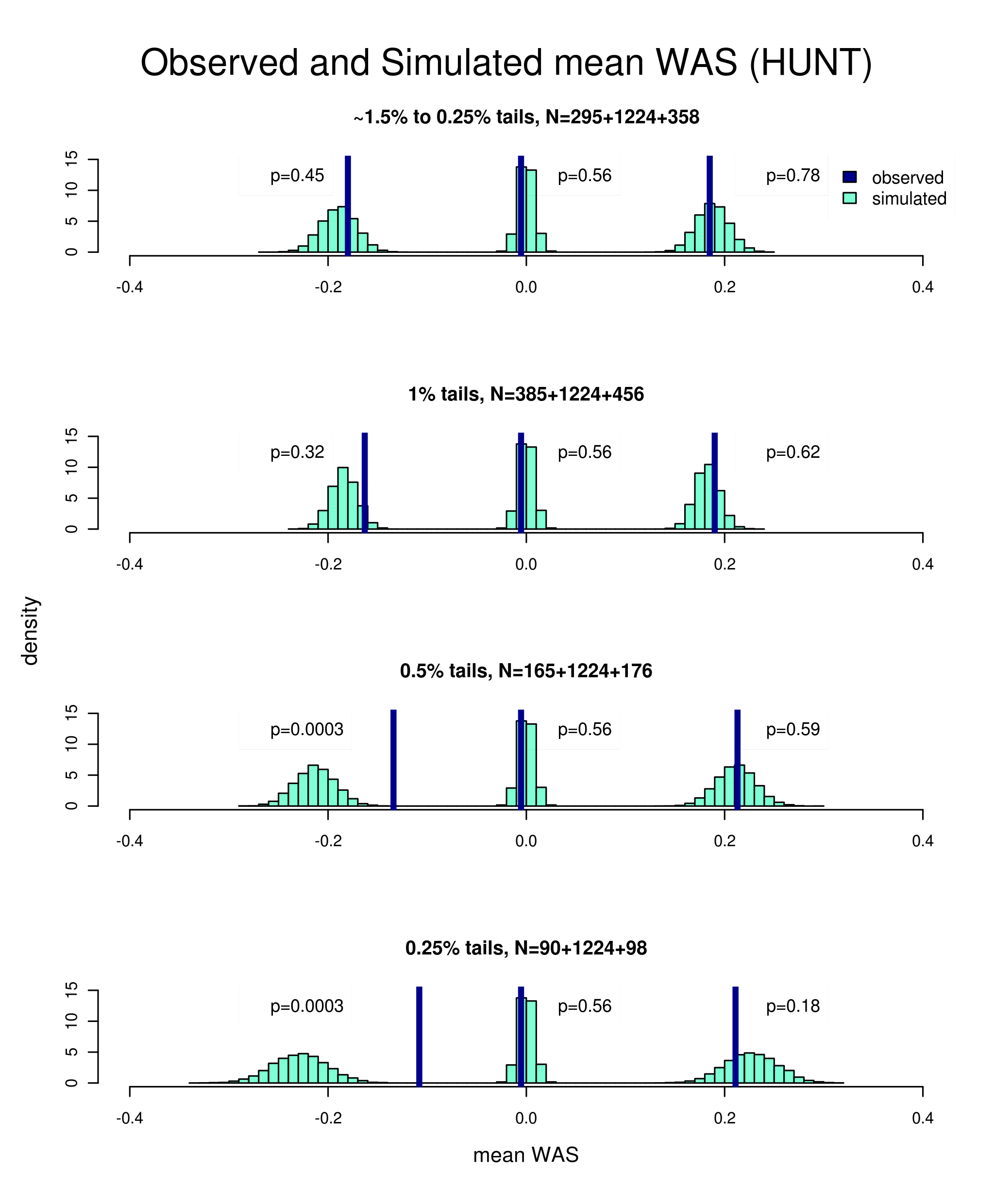

Supplement: Figure S2 — Comparison of the observed versus simulated mean weighted allele score (WAS) in the HUNT study. The plot shows the result of comparing the mean WAS of the short and tall individuals observed in the HUNT cohort against that obtained from simulation. Each row represents a different stratification of the extremes identical to those defined in Figure 2. The plot also show the mean WAS of 1224 non-extreme individuals taken from the middle of the height distribution. There is no difference between the mean WAS of the non-extreme individuals from that obtained from simulation (p = 0.56). (TIF) [file pgen.1002439.s002.tif]

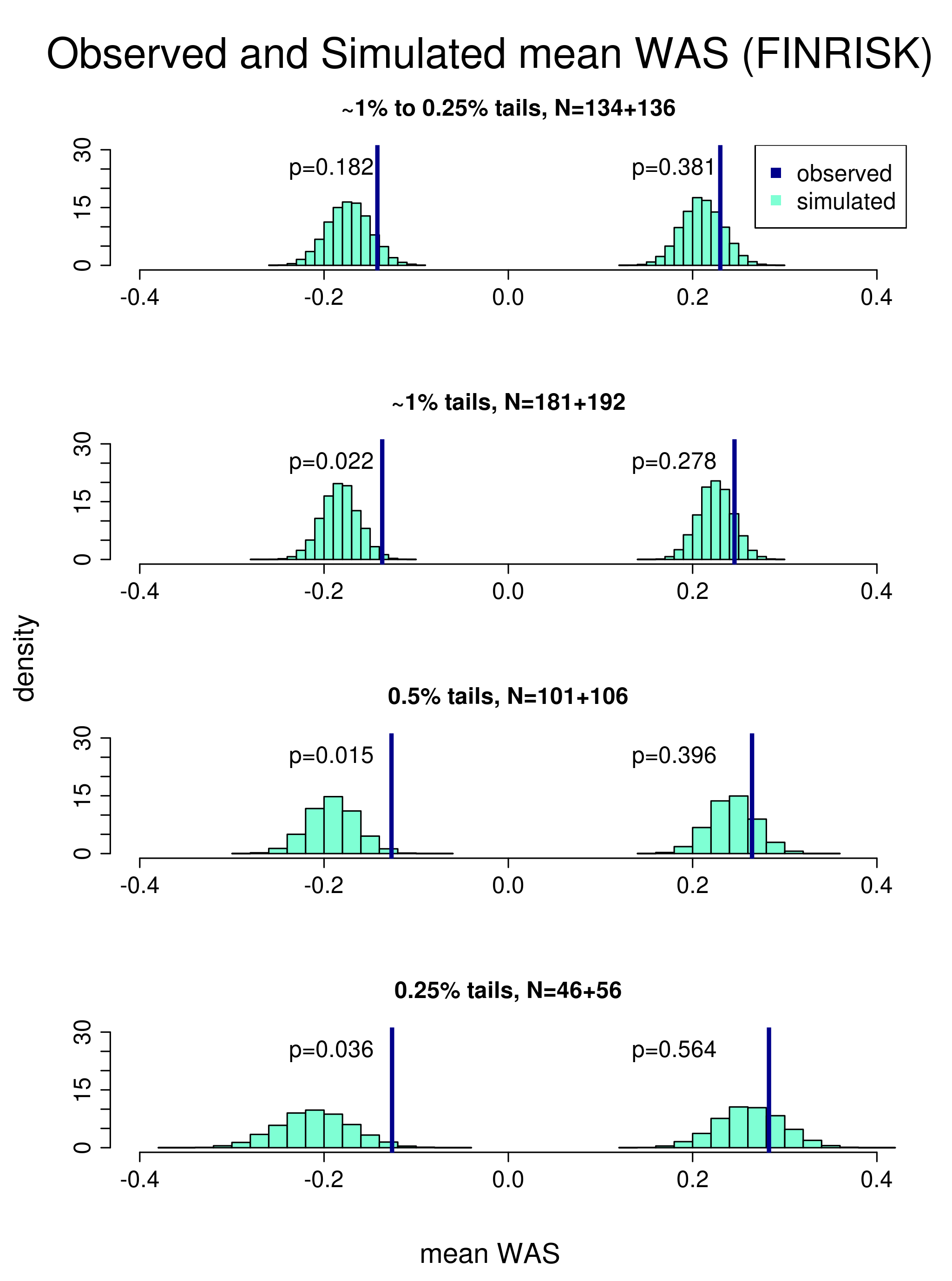

Supplement: Figure S3 — Comparison of the observed versus simulated mean weighted allele score (WAS) in the FINRISK study The plot shows the result of comparing the mean WAS of the short and tall individuals observed in the FINRISK cohort against that obtained from simulation. Each row represents a different stratification of the extremes identical to those defined in Figure 2. (TIF) [file pgen.1002439.s003.tif]

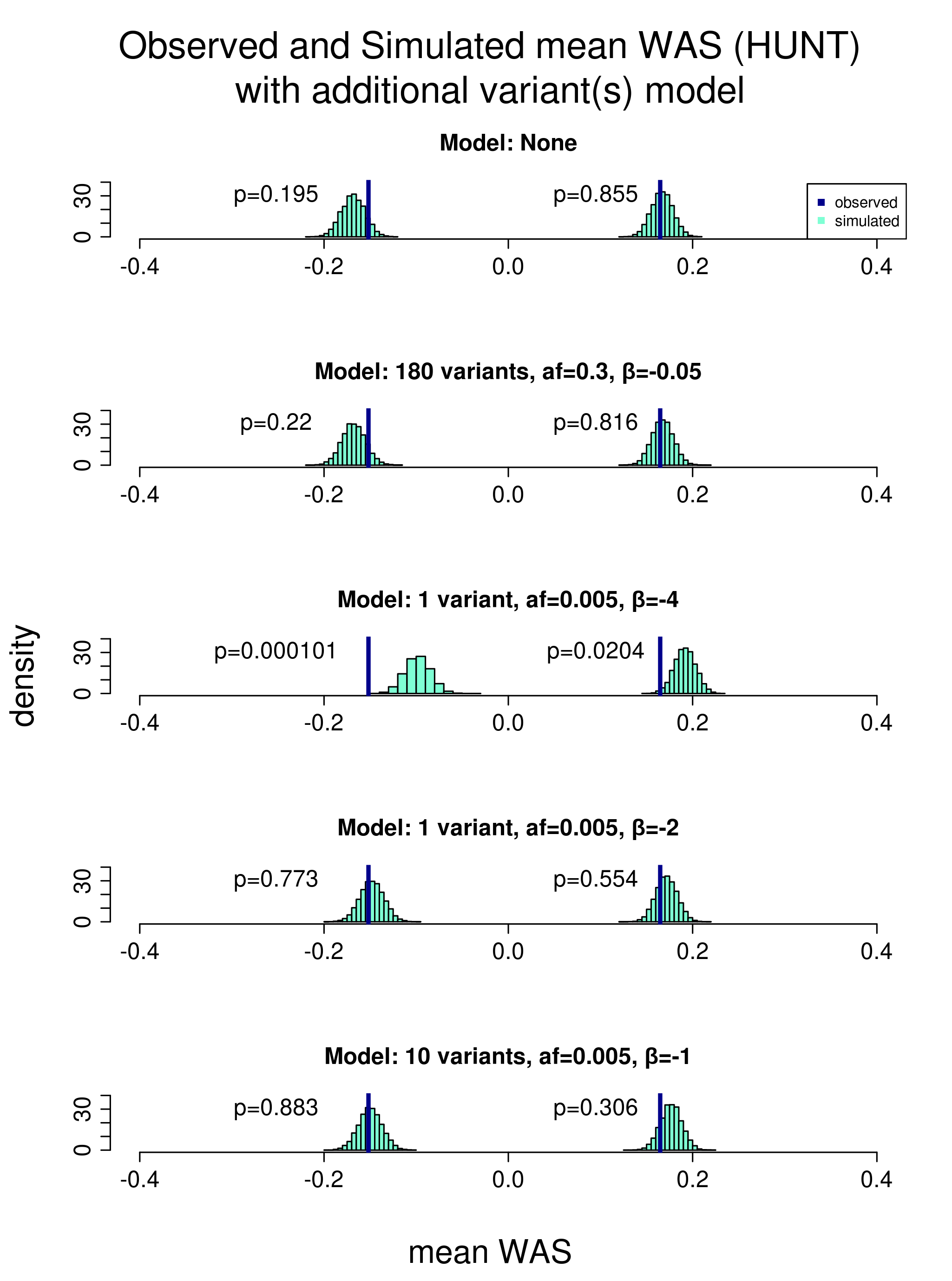

Supplement: Figure S4 — Comparison of the observed versus simulated mean WAS by incorporating additional variants (HUNT only). The plot shows the result of comparing the mean WAS of the short and tall individuals observed from only the HUNT cohort against that obtained from simulation with different scenarios of additional variants. Each row represents a different scenario identical to those defined in Figure 3. (TIF) [file pgen.1002439.s004.tif]

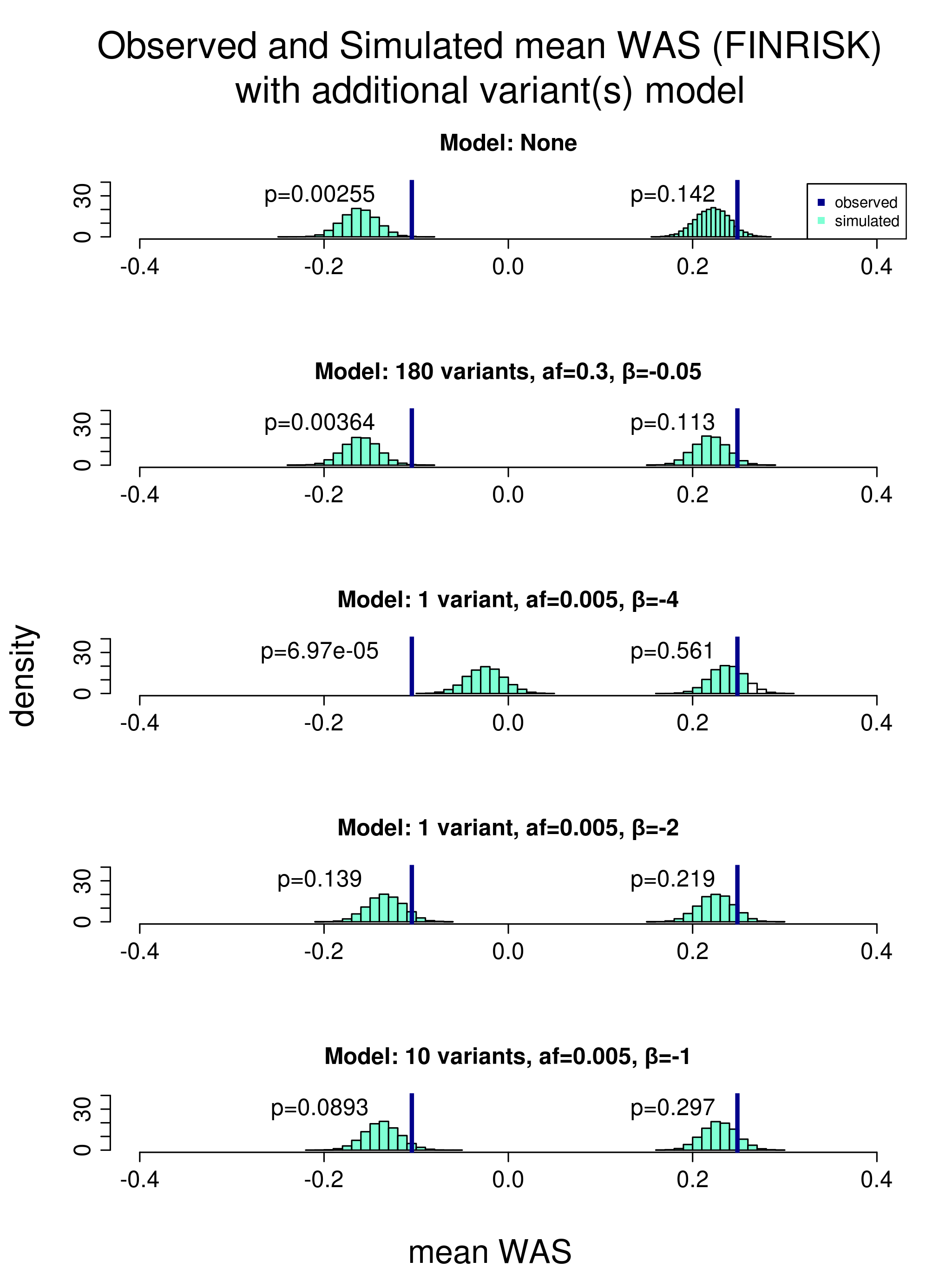

Supplement: Figure S5 — Comparison of the observed versus simulated mean WAS by incorporating additional variants (FINRISK only). The plot shows the result of comparing the mean WAS of the short and tall individuals observed from only the FINRISK cohort against that obtained from simulation with different scenarios of additional variants. Each row represents a different scenario identical to those defined in Figure 3. (TIF) [file pgen.1002439.s005.tif]

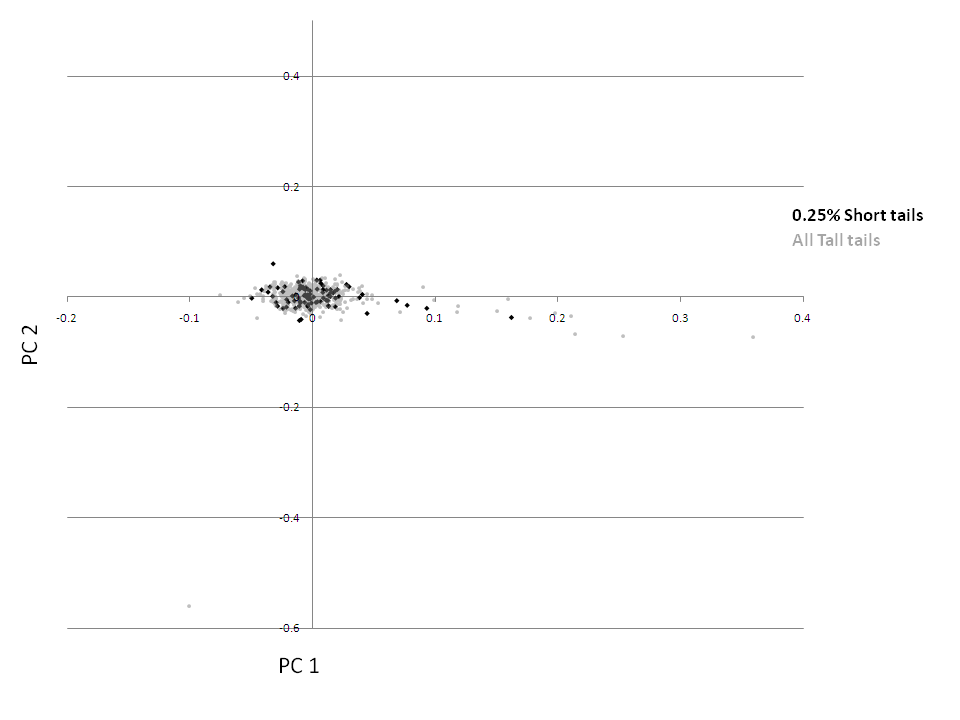

Supplement: Figure S6 — Principal component plots from the HUNT study for the 0.25% short individuals versus all tall individuals. The first two principal components obtained from Eigenstrat analysis for the samples in the HUNT study are plotted. There is no significant difference in principal components between the short and tall groups. (TIF) [file pgen.1002439.s006.tif]

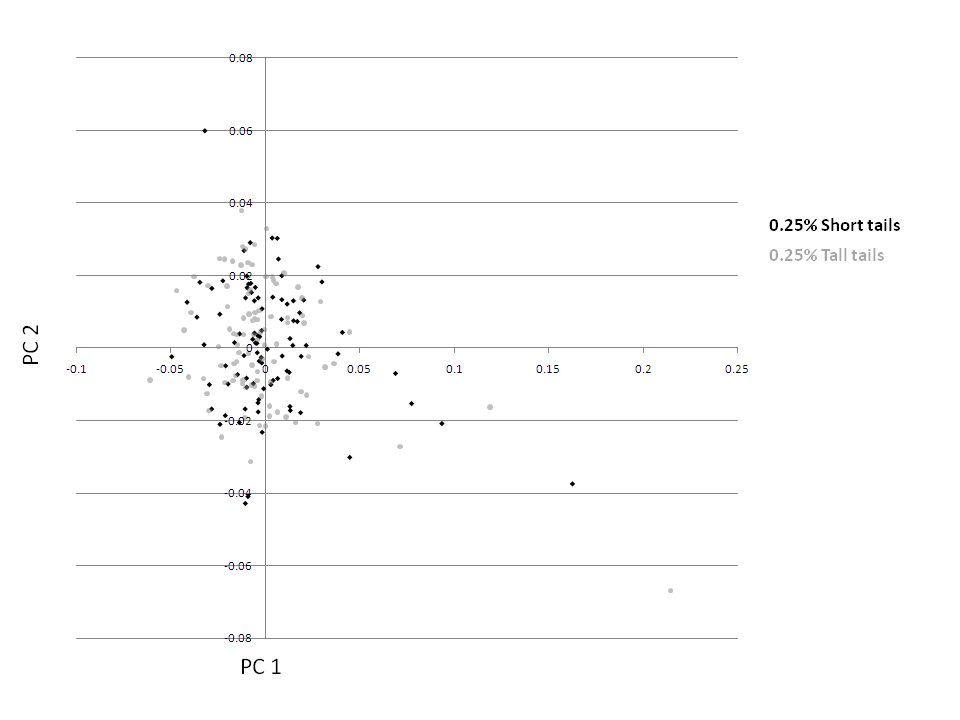

Supplement: Figure S7 — Principal component plots from the HUNT study for the 0.25% short vs. the 0.25% tall individuals. The first two principal components obtained from Eigenstrat analysis for the samples in the HUNT study are plotted. There is no significant difference in principal components between the short and tall groups. (TIF) [file pgen.1002439.s007.tif]
